# Phenology and Seed Yield Performance of Determinate Soybean Cultivars Grown at Elevated Temperatures in a Temperate Region

**DOI:** 10.1371/journal.pone.0165977

**Published:** 2016-11-03

**Authors:** Doug-Hwan Choi, Ho-Young Ban, Beom-Seok Seo, Kyu-Jong Lee, Byun-Woo Lee

**Affiliations:** 1 Department of Plant Science, College of Agriculture and Life Sciences, Seoul National University, Seoul, Korea; 2 Research Institute of Agriculture and Life Sciences, College of Agriculture and Life Sciences, Seoul National University, Seoul, Korea; College of Agricultural Sciences, UNITED STATES

## Abstract

Increased temperature means and fluctuations associated with climate change are predicted to exert profound effects on the seed yield of soybean. We conducted an experiment to evaluate the impacts of global warming on the phenology and yield of two determinate soybean cultivars in a temperate region (37.27°N, 126.99°E; Suwon, South Korea). These two soybean cultivars, Sinpaldalkong [maturity group (MG) IV] and Daewonkong (MG VI), were cultured on various sowing dates within a four-year period, under no water-stress conditions. Soybeans were kept in greenhouses controlled at the current ambient temperature (AT), AT+1.5°C, AT+3.0°C, and AT+5.0°C throughout the growth periods. Growth periods (VE–R7) were significantly prolonged by the elevated temperatures, especially the R1–R5 period. Cultivars exhibited no significant differences in seed yield at the AT+1.5°C and AT+3.0°C treatments, compared to AT, while a significant yield reduction was observed at the AT+5.0°C treatment. Yield reductions resulted from limited seed number, which was due to an overall low numbers of pods and seeds per pod. Heat stress conditions induced a decrease in pod number to a greater degree than in seed number per pod. Individual seed weight exhibited no significant variation among temperature elevation treatments; thus, seed weight likely had negligible impacts on overall seed yield. A boundary line analysis (using quantile regression) estimated optimum temperatures for seed number at 26.4 to 26.8°C (VE–R5) for both cultivars; the optimum temperatures (R5–R7) for single seed weight were estimated at 25.2°C for the Sinpaldalkong smaller-seeded cultivar, and at 22.3°C for the Daewonkong larger-seeded cultivar. The optimum growing season (VE–R7) temperatures for seed yield, which were estimated by combining the two boundary lines for seed number and seed weight, were 26.4 and 25.0°C for the Sinpaldalkong and Daewonkong cultivars, respectively. Considering the current soybean growing season temperature, which ranges from 21.7 (in the north) to 24.6°C (in the south) in South Korea, and the temperature response of potential soybean yields, further warming of less than approximately 1°C would not become a critical limiting factor for soybean production in South Korea.

## Introduction

Soybeans (*Glycine max* L. Merr.) are intensely cultivated worldwide due to the seeds containing high levels of oil and protein [1, 2]. Soybeans are native to Northeast Asia, including Korea [3], and are utilized in various products including tofu, soy sauce, bean paste, soybean oil, and soy milk.

Korea has experienced warming approximately twice as fast as the average rate of global warming, and the country is expected to experience continued warming in the future [4]. The average surface temperature of the Korean peninsula is projected to rise by 2.8 to 5.3°C by the end of this century, under RCP 4.5 and 8.5 scenarios, respectively [5]; warming is predicted to increase the average global surface temperature from 1.1 to 4.8°C, under the same scenarios [6]. The rise in temperature is expected to have substantial effects on the development and growth of crops, including soybeans [7].

Temperature is an important environmental factor that affects the development, growth, and yield of soybeans. Development rates accelerate when temperatures increase from below the optimum or decrease from above the optimum temperature [8]. Optimum temperatures were reported to be 30.0°C for vegetative development [9] and 26.0°C for anthesis [10]. In addition, the optimum temperature for reproductive development was reported to vary between 25.0 and 29.0°C, without significant differences between cultivars of differing maturity. However, a slight increasing trend was observed from V1 to R7 [11]. The optimum temperatures for soybean growth and total yield differed from the optimum temperatures for plant development. The maximum vegetative biomass of soybeans was achieved at temperatures ranging from 25.0 to 37.0°C [12]. The number of seeds and seed size are two main yield components of soybeans; the number of seeds is determined by the number of pods and number of seeds per pod [13]. Soybean yield components are negatively correlated and temperature-dependent [14, 15]. Compared with the optimum temperature for vegetative growth, the optimum temperatures for single seed growth (23.5°C) and seed size (23°C) of soybeans were lower as reported by Egli and Wardlaw [16]. Temporary exposure to extreme temperatures can also affect yield-determining processes. Short heat stress (6°C above ambient) only for 3 days reduced photosynthesis and increased oxidative stress that can cause yield loss in soybean under field condition [17]. Pollen viability (seed set) is decreased by exposure to instantaneous temperatures above 30.0°C in vitro [18]. Understanding how yield components respond to elevated temperatures is important for predicting soybean yields.

Several studies have examined the impacts of increasing temperatures associated with climate change on soybean seed production. Elevated temperatures caused lower net photosynthetic carbon assimilation and biomass production, which were attributed to declines in stomatal conductance and intercellular carbon dioxide and resulted in yield reduction [19]. Yield responses to temperature elevation (above current levels) vary according to different climate regions. In the southern United States, where growing season temperatures range from 25.0 to 27.0°C, soybean yields were predicted to decline with a temperature increase to 26.7°C. Conversely, soybean yields would increase in the upper Midwestern United States, with a growing season temperature of 22.5°C [20]. Increasing air temperature significantly reduced pod number, seed number, seed size, and seed yield in temperature gradient chambers that maintained up to approximately 3.0°C higher than 26.4°C, which is typical of growing season air temperatures in warm parts of Japan [21]. In addition, temperature stress was induced by a daily maximum temperature and a minimal night temperature of 38.0 and 28.0°C, respectively. These temperature regimes resulted in the abscission of flowers and pods, and a low pod set percentage [22]. However, the numbers of pods and seeds, and seed yield increased at elevated temperatures in Japanese regions, with a mean growing season temperature of approximately 21.0°C [23]. The soybean crop growth model CROPGRO-Soybean predicted that the highest grain yield would occur between the temperatures of 22.0 to 24.0°C, and a progressive yield decrease would result from plummeting temperatures [10, 12].

The mean air temperature of the soybean growing season (VE–R7) during the last ten years ranged from 21.7°C (northern region) to 24.6°C (southern region) in South Korea. According to the reported optimum temperature range of 22.0 to 24.0°C [10, 12], current soybean growing season temperatures are close to the optimum. Thus, further warming is anticipated to exceed the reported optimum temperature range and decrease soybean yields in southern regions of South Korea. However, few experimental studies have documented the impacts of climate change on soybean growth and yield in South Korea. Therefore, this study aimed to examine the effects of elevated air temperatures on the phenology, yield components, and yield of two determinate soybean cultivars in a temperate region of South Korea.

## Materials and Methods

### Plant materials and growth conditions

Two determinate soybean cultivars, Sinpaldalkong (MG IV) and Daewonkong (MG VI) [24], were pot-cultured (15 cm in diameter, 40 cm in height) in four sunlit temperature-controlled greenhouses (W × D × H; 5.0 × 14.0 × 3.5 m) at the Experimental Farm of Seoul National University, Suwon, South Korea (37°16´N, 126°59´E). To establish a soybean canopy similar to field conditions, the pots were arranged in four rows, where plants and rows were spaced 15 and 45 cm apart, respectively. Only the inner two rows were used for observing phenology, seed yield, and yield-related traits. A pot experiment was conducted, rather than a field experiment, to control soil water more precisely [25]. Although the absolute growth and yield performances tend to differ between field and pot experiments, the responses to environmental variables, such as temperature and soil water, are reported to exhibit similar trends [26, 27]. Greenhouses were controlled to targets of current ambient air temperature (AT), AT+1.5°C, AT+3.0°C, and AT+5.0°C using heating and ventilation systems linked to a CR1000 Measurement and Control Datalogger (Campbell Scientific Inc., Logan, UT, USA). In addition, the greenhouses were covered by polyethylene film with solar transmittance of approximately 60% (2009, 2013, and 2014) or 85% (2015). Sidewalls were removed from the AT treatment greenhouse to maintain outdoor ambient temperatures. Loam soil with a pH of 6.6, soil organic matter of 1.9%, and CEC of 15.4 cmol^(+)^/kg was utilized in the experiments, and chemical fertilizers were applied to each pot, which contained 0.08, 0.08, and 0.09 g of N, P_2_O_5_, and K_2_O, respectively. The Sinpaldalkong cultivar was sown on: May 31, 2009; June 15, 2009; June 15, 2013; June 30, 2013; May 30, 2014; June 20, 2014; and June 15, 2015. The Daewonkong cultivar was sown on: May 31, 2009; June 15, 2009; June 20, 2014; July 11, 2014; and June 20, 2015. After emergence from the soil, the plants were thinned to one (2013, 2014, and 2015) or two (2009) plants per pot, and then exposed to no water-stress conditions by sub-irrigation. Weeding and pesticide applications were carried out when necessary.

### Measurements of phenology, yield, and weather

The developmental stages, as described by Fehr et al. [28], were surveyed every two to three days, and then the following was recorded: date of emergence (VE), beginning of flowering (R1), beginning of seed filling (R5), and physiological maturity (R7). The number of newly opened flowers was recorded every day during the flowering period from four pots only in 2015. Plant components observed above ground were harvested at maturity (R8) from five pots in 2013, and from eight pots in 2009, 2014, and 2015. The numbers of fertile pods and seeds, total fertile seed weight, and seed moisture content of each pot were measured to calculate the yield and components per pot. The single seed weight and seed yield per pot were adjusted to a moisture content of 15%. Air temperature and solar radiation values in each greenhouse were recorded (per minute) using a CR1000 data logger equipped with a platinum resistor thermoprobe housed in a naturally ventilated, six-plate radiation shield (Campbell Scientific), and a pyranometer (Kipp & Zonen, Delft, Netherlands), respectively. Outdoor air temperature and solar radiation values were obtained from the Suwon Meteorological Station, which is located approximately 0.5 km from the experimental greenhouses.

### Data analysis

Outliers identified by median quantile regression (with a cut-off value of 3.0) in scatter plots of seed yield and yield components versus mean air temperature, during the specific growth period of each cultivar, were eliminated. Development rates were calculated as the inverse of the number of days between two developmental stages. Pod set ratio of each pot was determined by dividing the number of fertile pods at maturity by the total number of opened flowers. To evaluate the effects of elevated temperature on phenology, seed yield, and yield components, analysis of variance (ANOVA) and Duncan’s multiple range test for significant differences between the treatment means were performed. A correlation and path analysis (Covariance Analysis of Linear Structural Equations: CALIS) was conducted to identify relationships between seed yield and its components, and to determine the direct contribution of yield components and yield-related components to yield and yield components, respectively. A boundary line analysis [29], which can exclude experimental errors and the effects of limiting factors [30], was employed to delineate the responses of seed yield components to mean air temperatures measured during: the VE–R5 period to calculate seed number per pot, the R5–R7 period to calculate single seed weight, and the VE–R7 period to calculate seed yield. The boundary line parameters of seed number and single seed weight were estimated by a quadratic quantile regression (95^th^ percentile). The boundary line response of potential yield to whole growing season temperature was obtained by combining those two boundary lines for yield components. Furthermore, mean growing season temperatures were calculated as the average of the mean temperature of VE–R5 and R5–R7 weighted by the durations of the two periods. Statistical analyses were performed using the statistical analysis software SAS Version 9.4 (SAS Institute Inc., Cary, NC, USA).

## Results

### Meteorological conditions

**Fig 1:**
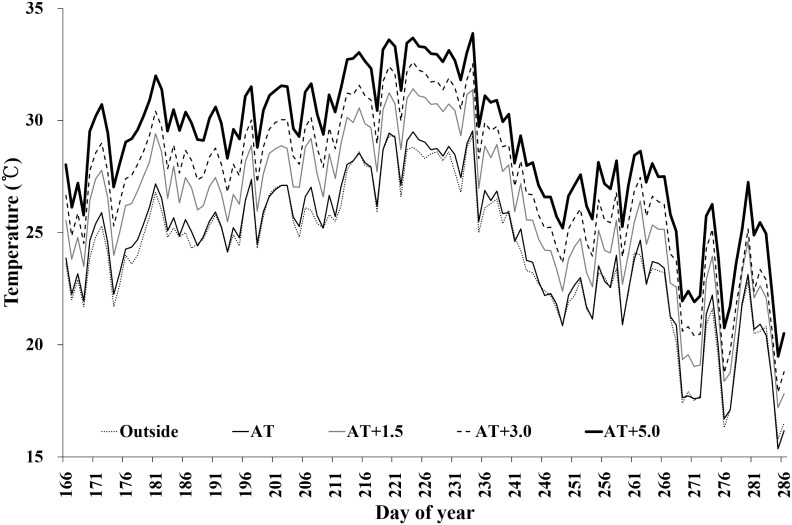
Daily mean air temperatures in the experimental greenhouses that were controlled to target temperatures of AT, AT+1.5°C, AT+3.0°C, and AT+5.0°C during the growing seasons. Only data collected in 2013 are presented as an example of seasonal temperature variation. Dotted gray, continuous black, continuous gray, dashed black, and bold black lines indicate the temperatures of outside, AT, AT+1.5°C, AT+3.0°C, and AT+5.0°C, respectively.

The four-month mean air temperature in the AT ranged from 23.5 to 25.1°C during the experiments; the four-month mean temperatures of AT+1.5°C, AT+3.0°C, and AT+5.0°C were 1.5–1.8°C, 2.7–3.0°C, and 4–4.6°C higher than the AT, respectively (Table 1). The monthly mean air temperatures in the ATs throughout the experimental periods were similar to outdoor air temperatures, with any observed differences ranging within ±1.0°C. Fig 1 presents the daily changes in mean air temperatures within the temperature-controlled greenhouses during the 2013 growing season. Differences between the temperature treatments were consistently maintained during the growing seasons.

**Table 1:** Monthly mean air temperature and solar radiation values in the experimental greenhouses that were controlled to target temperatures of AT, AT+1.5°C, AT+3.0°C, and AT+5.0°C during the growing seasons over the four-year period.

		June	July	August	September	Four-month mean
Air temperature (°C)						
2009	Outside	22.1	24.2	25.7	21.6	23.4
	AT	22.1	24.2	25.8	21.6	23.5
	AT+1.5°C	23.9	26.0	27.5	23.3	25.2
	AT+3.0°C	25.0	27.0	28.6	24.6	26.3
	AT+5.0°C	26.4	28.7	30.6	26.4	28.1
2013	Outside	23.5	25.5	27.4	21.7	24.6
	AT	23.9	25.8	27.7	21.9	24.8
	AT+1.5°C	25.6	27.5	29.5	23.5	26.6
	AT+3.0°C	26.8	28.7	30.7	24.8	27.8
	AT+5.0°C	28.8	30.3	31.9	26.3	29.2
2014	Outside	22.9	25.6	24.7	21.8	23.8
	AT	22.9	25.6	24.7	21.8	23.8
	AT+1.5°C	24.6	27.2	26.1	23.3	25.3
	AT+3.0°C	25.6	28.4	27.3	24.5	26.5
	AT+5.0°C	27.2	29.9	28.2	25.8	27.8
2015	Outside	23.1	25.5	26.2	22.1	24.2
	AT	24.0	26.3	27.1	22.8	25.1
	AT+1.5°C	25.4	27.7	28.5	24.2	26.5
	AT+3.0°C	26.9	29.2	29.0	25.5	27.7
	AT+5.0°C	28.8	31.0	31.6	27.3	29.7
Solar radiation (MJ/m^2^·day)						
2009	Inside the greenhouse	11.7	9.8	10.4	10.1	10.5
2013		11.5	6.0	9.4	8.4	8.8
2014		11.0	9.4	7.7	8.1	9.0
2015		14.5	11.5	11.4	11.3	12.2

### Phenology responses to elevated temperature

**Fig 2:**
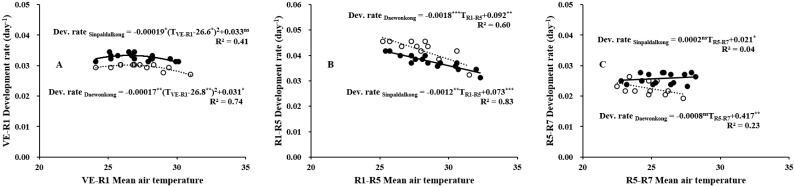
Temperature responses of development rates during the VE–R1 (A), R1–R5 (B), and R5–R7 (C) periods of the Sinpaldalkong (filled circles and continuous lines) and Daewonkong (open circles and dashed lines) cultivars planted during the typical sowing season (mid-June). Plotted points represent the mean values of the temperature treatments during each growing season. ***, **, *, and ns denote significance of the regression coefficients at p < 0.001, p < 0.01, p < 0.05, and not significant (p > 0.05), respectively.

Phenophase durations of the soybean cultivars planted during the typical sowing season (mid-June) under different temperature treatments are shown in Table 2. The Sinpaldalkong mid-maturing cultivar had a significantly shorter growing period than the Daewonkong late-maturing cultivar. According to ANOVA, above ambient temperatures had no significant effects on the duration of emergence (VE) to flowering (R1). Although the R1–R5 and R5–R7 periods did not differ significantly among the temperature treatments, the post-anthesis period (R1–R7) was significantly prolonged by temperature elevation, without the temperature × cultivar interaction. Compared with the AT, the R1–R7 period of the AT+5.0°C treatment was 4 and 7 days longer for the Sinpaldalkong and Daewonkong cultivars, respectively. Regardless of cultivar type, the rate of development to flowering exhibited a quadratic response to temperature, with an optimum temperature of approximately 26.7°C (Fig 2). The development rate during the R1–R5 period decreased linearly with a rise in temperature from 25.2 to 32.3°C. The slopes of the regression lines between the mean temperature and the development rate during the R5–R7 period were not significant in both cultivars, indicating that the development rate during seed filling was not affected by a temperature increase from 22.8 to 27.9°C in both cultivars.

**Table 2:** Phenology responses of two determinate soybean cultivars with different maturity groups planted during the typical sowing season (mid-June) to temperature treatments that were controlled to target temperatures of AT, AT+1.5°C, AT+3.0°C, and AT+5.0°C.

Cultivar and temperature treatment	Days between phenophases				
	VE–R1	R1–R5	R5–R7	R1–R7	VE–R7
**Sinpaldalkong (MG IV)**					
AT	30.5	25.3	38.8	64.0b	94.5b
AT+1.5°C	29.8	26.3	38.8	65.0b	94.8b
AT+3.0°C	30.8	27.0	39.0	66.0b	96.8b
AT+5.0°C	31.5	28.8	39.5	68.3a	99.8a
**Cultivar mean**	30.6	26.8	39.0	65.8	96.4
**Daewonkong (MG VI)**					
AT	33.7	22.7	42.3	65.0b	98.7b
AT+1.5°C	33.0	23.7	45.0	68.7ab	101.7ab
AT+3.0°C	34.3	24.3	45.0	69.3ab	103.7ab
AT+5.0°C	34.7	26.0	46.0	71.7a	106.3a
**Cultivar mean**	33.9	24.2	44.6	68.7	102.6
**Temp. treatment mean**					
AT	31.9	24.1	40.3	64.4c	96.3c
AT+1.5°C	31.4	25.1	41.4	66.6bc	97.7bc
AT+3.0°C	32.3	25.9	41.6	67.4b	99.7b
AT+5.0°C	32.9	27.6	42.3	69.7a	102.6a
**ANOVA results**					
Temperature (T)	ns	ns	ns	***	***
Cultivar (C)	***	**	**	**	***
T × C	ns	ns	ns	ns	ns

Values followed by the same letters within a column for each cultivar and temperature treatment mean were not significantly different at the confidence level of 5%, as determined by Duncan’s multiple range tests. ***, **, and ns denote significance at p < 0.001, p < 0.01, and not significant (p > 0.05), respectively.

### Yield and yield component responses to elevated temperatures

Seed yield and yield components of the Sinpaldalkong and Daewonkong cultivars affected by the temperature elevation treatments (planted during the typical sowing season) are shown in Table 3. The Sinpaldalkong cultivar had a smaller seed size and a higher seed number than the Daewonkong cultivar. There were no significant temperature × cultivar interactions for seed yield and yield components. The cultivars exhibited no significant differences in seed yield from the AT+1.5°C and AT+3.0°C treatments, compared with the AT, but a significant yield reduction was observed for the AT+5.0°C treatment. Pod number, number of seeds per pod, and number of seeds per pot increased with temperature increases of 3.0°C, 1.5°C, and 3.0°C above ambient temperature, respectively; all numbers decreased with further temperature elevation. Single seed weight tended to decrease slightly with increases in temperature; however, no significant differences were detected between the different temperature treatments.

**Table 3:** Responses of seed yield and yield components to temperature treatments that were controlled to target temperatures of AT, AT+1.5°C, AT+3.0°C, and AT+5.0°C in two determinate soybean cultivars with different maturity groups (MGs) planted during the typical sowing season (mid-June).

Cultivar and temperature treatment	Pod number (pot^-1^)	Seed number (pod^-1^)	Seed number (pot^-1^)	Single seed weight (mg)	Seed yield (g pot^-1^)
**Sinpaldalkong (MG IV)**					
AT	84.1a	2.07ab	174a	196	33.4a
AT+1.5°C	85.3a	2.11a	180a	187	33.0a
AT+3.0°C	95.3a	1.97b	186a	184	33.8a
AT+5.0°C	67.2b	1.84c	130b	194	24.7b
**Cultivar mean**	81.0	1.99	163	191	30.5
**Daewonkong (MG VI)**					
AT	71.5b	1.87a	134b	267	34.7ab
AT+1.5°C	81.4a	1.89a	155a	255	38.0a
AT+3.0°C	84.1a	1.85ab	155a	249	37.2a
AT+5.0°C	68.9b	1.79b	127b	249	32.0b
**Cultivar mean**	75.6	1.85	141	255	35.1
**Temp. treatment mean**					
AT	78.3ab	1.98ab	156a	228	34.0a
AT+1.5°C	83.5a	2.01a	168a	218	35.3a
AT+3.0°C	90.1a	1.91b	172a	214	35.4a
AT+5.0°C	68.0b	1.81c	129b	219	28.0b
**ANOVA results**					
Temperature (T)	**	***	***	ns	**
Cultivar (C)	ns	***	**	***	**
T × C	ns	ns	ns	ns	ns

Values followed by the same letters within a column for each cultivar and temperature treatment mean were not significantly different at a confidence level of 5%, as determined by Duncan’s multiple range tests. ***, **, and ns denote significance at p < 0.001, p < 0.01, and not significant (p > 0.05), respectively.

**Fig 3:**
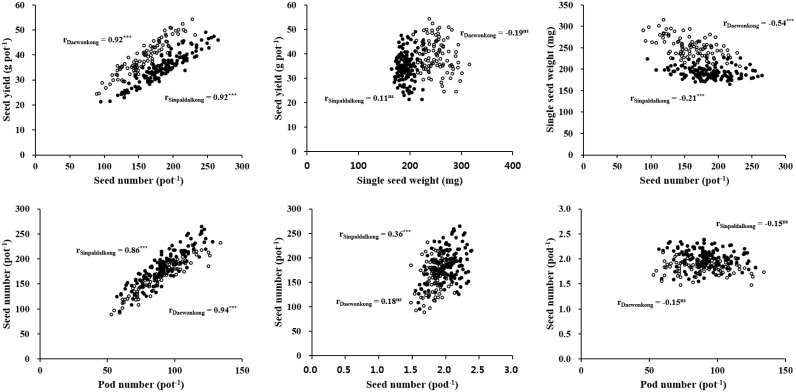
Correlations between seed yield and yield components of the Sinpaldalkong (filled circles) and Daewonkong (open circles) cultivars grown under elevated temperature conditions with different sowing dates over a four-year period. *** and ns denote significance at p < 0.001 and not significant (p > 0.05), respectively.

Correlations between seed yield and yield components in two determinate soybean cultivars, Sinpaldalkong and Daewonkong, are presented in Fig 3. Seed yield showed highly significant positive correlations (r = 0.92***) with seed numbers per pot in both cultivars; no significant correlations with single seed weight were observed. Seed number per pot showed highly significant positive correlations (r = 0.86*** and 0.94***) with pod numbers per pot in the Sinpaldalkong and Daewonkong cultivars, respectively; a significantly positive correlation with the number of seeds per pod was observed only for the Sinpaldalkong cultivar (r = 0.36***). Single seed weight showed significantly negative correlations (r = −0.21*** and −0.54***) with seed number per pot for the Sinpaldalkong and Daewonkong cultivars, respectively.

**Fig 4:**
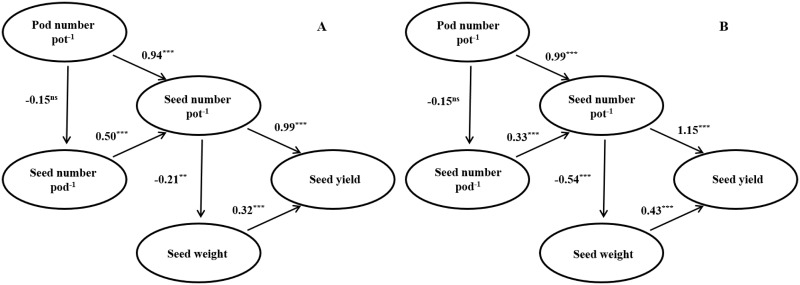
Path diagram: the direct contribution of yield components to yield, pod number per pot and seed number per pod to seed number per pot, and seed number per pot to single seed weight in two soybean cultivars, Sinpaldalkong (A) and Daewonkong (B), grown under elevated temperature conditions with different sowing dates over a four-year period. ***, **, and ns denote significance at p < 0.001, p < 0.01, and not significant (p > 0.05), respectively.

Path analyses were conducted according to the predetermined causal relationships between seed yield and yield components (Fig 4). Regardless of cultivar type, the seed yield variation was more closely associated with seed number per pot, rather than with the single seed weight. Pod number per pot showed significant association with the variation in seed number per pot, rather than with the seed number per pod. Pod number exerted negligible effects on the seed number per pod. Furthermore, the seed number per pot negatively affected single seed weight.

### Boundary line response of the yield and yield components to temperature

**Fig 5:**
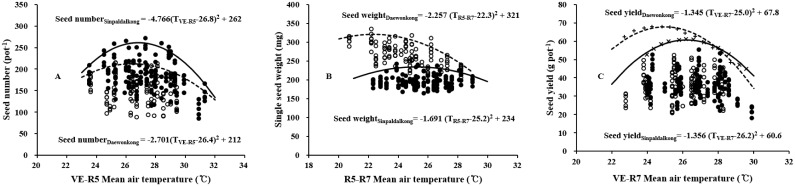
Boundary line analysis of seed number (A), single seed weight (B), and seed yield (C) responses to mean air temperatures during specific growth periods for two determinate soybean cultivars, Sinpaldalkong and Daewonkong. Filled and open circles represent the data for the Sinpaldalkong and Daewonkong cultivars, respectively. In A and B, each continuous and dashed line indicates the 95% quantile regression lines for the Sinpaldalkong and Daewonkong cultivars, respectively. In C, the cross and plus symbols indicate potential yields, which were calculated by multiplying the boundary points of the seed number by the single seed weight for the Sinpaldalkong and Daewonkong cultivars, respectively. Continuous and dashed lines represent the quadratic regression lines of potential yield points for the Sinpaldalkong and Daewonkong cultivars, respectively.

Temperature responses of plant growth and development are characterized by cardinal temperatures: minimum, optimum, and maximum temperatures. Cardinal temperatures vary among genotypes and growth stages, even within the same genotype. The boundary lines of seed number and single seed weight in relation to mean air temperature (during specific growth periods when those traits are determined) were well fitted to the quadratic equations, revealing an optimum temperature (Fig 5). Seed number and single seed weight were well fitted to the mean a during the VE–R5 and R5–R7 periods, respectively. Optimum temperature ranges were estimated to be 26.8 and 26.4°C for seed number, and 25.2 and 22.3°C for single seed weight for the Sinpaldalkong and Daewonkong cultivars, respectively. Optimum temperatures for seed number were similar for both cultivars, whereas the optimum temperature for single seed weight was lower for the Daewonkong large-seeded cultivar than for the Sinpaldalkong cultivar. According to the boundary line response of potential seed yields to growing seasons (VE–R7), the optimum mean growing season temperature (which was established by combining the boundary responses of the two yield components) for the potential seed yield were estimated to be 26.2 and 25.0°C for the Sinpaldalkong and Daewonkong cultivars, respectively. The optimum temperature for seed yield was lower for the Daewonkong cultivar, due to the lower optimum temperature for single seed weight.

## Discussion

### Phenology responses to elevated temperatures

Owing to the short-day nature of soybean plants, the onset of flowering (R1) is primarily affected by the photoperiod and temperature prior to flowering [31–34]. No significant effects on flowering time (VE–R1) were observed for the two cultivars, which were grown under similar photoperiod conditions and various temperature treatments (Table 2). This indicates that temperature (VE–R1) variation within the range from 24.1 to 31.0°C, which is very close to the reported optimum temperature range of 25.0 to 29.0°C for flower induction in soybeans [10, 11], exerted negligible effects on the development rate during the growth period (Fig 2). The developmental rate during this period exhibited a small quadratic response to mean growth temperatures, with a maximum rate occurring at approximately 27.0°C. The post-flowering duration (R1–R7) was lengthened with elevated temperatures, mainly due to the increased duration of R1–R5. Growth temperatures during the R1–R5 period increased with the temperature elevation treatments within the range of 25.2 to 32.3°C (Fig 2); this surpassed the optimum temperature (21.5°C) for the post-flowering (R1–R7) development rate [34]. These supra-optimum temperatures should have slowed the post-flowering development increasingly with temperature elevation above ambient, which would have subsequently lengthened the post-flowering period (Fig 2). Tacarindua et al. [21] reported similar results, where increasing air temperatures had no effect on the flowering time (R1), but the pod set period (R1–R5) was significantly increased by the increasing air temperatures above 26.4°C (average temperature for the entire growing season) for MG IV cultivars.

### Yield and yield component responses to elevated air temperatures

**Fig 6:**
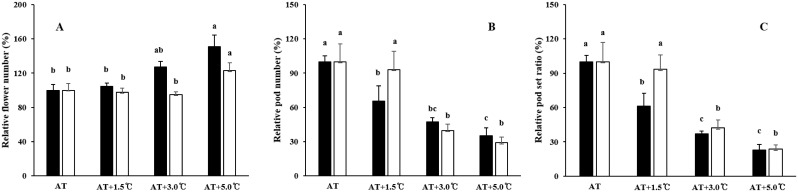
The numbers of flowers (A), pods (B), and the pod set ratio (C) presented as relative values to the AT for the Sinpaldalkong (filled rectangles) and Daewonkong (open rectangles) cultivars, grown under four different temperature conditions in 2015. The error bars represent the standard error of the mean. The same letters above the bars within each cultivar indicate no significant differences between the temperature treatments, as determined by Duncan’s multiple range test at a confidence level of 5%.

Pod number and seed number per pot increased with temperatures elevation up to 3.0°C above AT treatment, and then decreased with further temperature elevation (Table 3). Seed number is determined by the number of pods and the number of seeds per pod [13]. Increased flowering and pod set (R1–R5) periods (due to temperature increases) may lead to higher pod numbers in the AT+1.5°C and AT+3.0°C treatments. Increases in pod numbers are likely attributable to increased flower numbers, rather than the pod set ratio, as the flower number increased and the pod set ratio decreased with temperature elevation treatments above the outside ambient temperature in 2015 (Fig 6). Temperatures were abnormally high during the 2015 flowering period. The number of fertile pods is reported to be associated with the numbers of aborted and abscised flowers and pods, which occur due to heat stress during the R1–R5 period [35]. The daily mean temperature of approximately 25.0°C during the early reproductive stages favored a higher pod set than temperatures below 25.0°C [36]; temperatures exceeding a day temperature of 30.0°C resulted in decreased pod numbers [37]. Considering the temperature conditions during the R1–R5 period, which ranged between 25.2 and 32.3°C in our study (Fig 2), the high temperatures likely imposed adverse effects on pod sets at the AT+3.0°C and AT+5°C treatments. Seed number per pot and pod number per pot showed similar responses to temperature, as the variation in seed number per pot was predominantly determined by the pod number (Fig 4).

The number of seeds per pod significantly decreased with increases in temperatures above AT+1.5°C in our study. However, the number of seeds per pod was reported to be the yield component least affected by environmental conditions, including temperature [38]; this number is regarded as a fixed value of the genotype coefficient in the CROPGRO-Soybean model [39]. The number of seeds per pod is a function of the number of ovules per pod and the success of a seed set. Soybean ovaries contain one to four ovules, and the number of seeds averages between two and three per pod [40]. The number of seeds per pod is reduced by seed set failure, due to unfertilized ovules and the abortion of fertilized ovules from environmental stress, such as high temperatures during flowering and early seed developmental stages [35, 40, 41].

Maximum seed size is determined genetically, whereas the actual seed size is affected by seed number and environmental conditions during seed filling [42]. The optimum temperature for single seed growth and size is reported to be approximately 23.0°C [16]. An increase in temperature above the reported optimum from 22.5 to 28.2°C during the R5–R7 period (Fig 2) had no significant effects on single seed weight (Table 3). This result would be ascribed to the compensation effect of seed number on single seed weight. As in Table 3, seed number per pot increased with temperatures elevation up to 3.0°C above AT treatment, and then decreased with further temperature elevation. For both cultivars, single seed weight showed significant negative correlations with seed number per pot (Fig 3), indicating that single seed weight decreased from its maximum value to compensate for the increase in seed number per pot [38, 43].

Our results demonstrate that seed yield variations due to different temperature treatments, sowing dates, and experimental years were more closely associated with seed number variations, rather than with seed weight variations (Figs 3 and 4). Seed number had a strong association with the variation in pod number. The reproductive processes were impeded by high temperatures during early pod developmental stages, which led in lower seed yield despite a rapid physiological recovery from the heat stress [17]. Excluding the temperature elevation treatment of AT+5.0°C, our results revealed that a temperature increase within the range of 22.8 to 28.6°C (data not shown) during the whole growth period (VE–R7) had no significant effects on seed yield.

### Boundary line responses of yield and yield components to temperature

Seed number and individual seed weight are two main yield components of soybeans [13]. Optimum temperatures for seed number and seed weight were estimated using the boundary line analysis method first suggested by Webb [29], where it was frequently utilized for analyzing agronomic data obtained from field experiments where all factors could not be strictly controlled [44, 45]. The boundary line method involves fitting a line through the boundary points that appear in the uppermost section of the data scatter plot, as opposed to fitting regression lines through scattered data points. For a large data set, the boundary line is assumed to represent the maximum potential expression of a trait in response to a predictor variable, and in the absence of other limiting factors [30]. That is, the boundary lines in Fig 5 represent the potential maximum responses of yield components and yield to the mean temperatures during the phenophases (when those traits are determined). The estimated optimum temperatures are also the temperatures at which maximum expression is expected, in the absence of other limiting factors.

Seed number is affected by flower differentiation, pod setting, pod abortion, ovule differentiation, and ovule abortion. The number of differentiated flowers is affected by the degree of vegetative growth prior to flowering, as flowers can be produced on a plant’s main stem and on branch nodes [46]. Flowering ends and plants reach their maximum vegetative weight at the beginning of the R5 period [47]. Abscission and abortion of flowers, pods, and fertilized ovules occur due to heat stress experienced during the R1–R5 period [35]. Accordingly, the seed number is affected by temperature conditions from emergence (VE) to the R5 stage. The optimum temperature for seed number estimated at approximately 26.5°C during the VE–R5 period for both cultivars (Fig 5) is an appropriate value upon consideration of the reported temperature responses for seed number components (as previously discussed). Moreover, in temperature gradient chamber experiments featuring the MG IV cultivar, the seed number was found to increase with growth temperature increases ranging from 20.0 (current) to 27.0°C in a cool region [23], and decrease with temperatures above the current growth temperature of 26.4°C in a warm region [21].

Although single seed weight did not significantly differ among the temperature treatments, the optimum temperatures for single seed weight were found using a boundary line analysis. Seed weight was primarily affected by the temperature during the seed filling period [48]. Optimum temperatures for soybean seed size and seed growth rate were reported to be 23.0°C [16, 38] and 23.5°C [16], respectively. These reported optimum temperatures differed slightly from our results; the optimum temperatures during the seed filling duration (R5–R7) for single seed weight were estimated at 25.2°C for the Sinpaldalkong (medium seed size) cultivar, and at 22.3°C for the Daewonkong (large seed size) cultivar (Fig 5). Evidence demonstrating that the optimum temperature for seed weight should differ according to the cultivar seed size has not yet been documented. However, plausible indirect evidence is available. A small-seeded soybean variety was reported to be less sensitive to yield components (including seed weight), resulting in deterioration due to high-temperature stress, compared with large-seeded genotypes [35]. Sexton et al. [49] also reported similar results, where small-seeded cultivars were found to be more advantageous than large-seeded ones, with regard to seed growth rate. In another leguminous crop, the chickpea, cultivars with small seed sizes were also found to have a higher heat tolerance than large-seeded cultivars [50]. These findings provide indirect evidence for crops other than soybeans, and thus further studies should document genotypic differences in the temperature responses of seed weight and seed yield.

**Fig 7:**
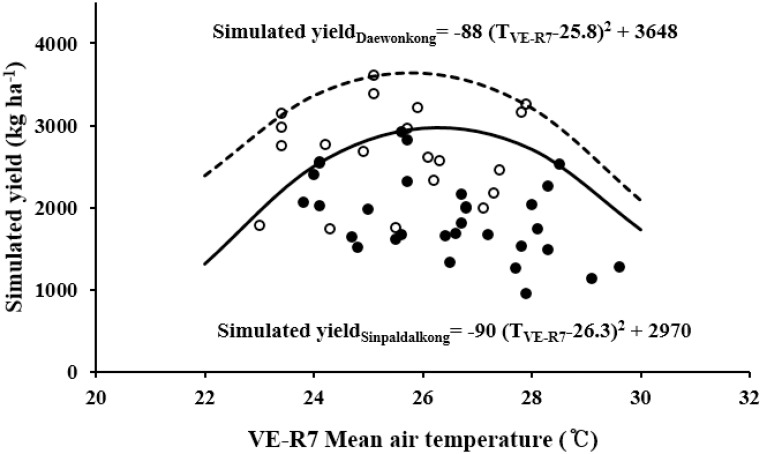
Boundary line analysis of temperature responses in simulated soybean yields using the CROPGRO-Soybean model for Sinpaldalkong (filled circles) and Daewonkong (open circles) cultivars, grown under elevated temperatures with different sowing dates over a four- year period. Genetic coefficients were calibrated with field experiment data independent from this experiment; soil characteristic data from the pot experiments were used; weather data were obtained inside the experimental greenhouses, and cultivation methods were used for the simulations. Continuous and dashed lines indicate the 95% quantile regression lines for the Sinpaldalkong and Daewonkong cultivars, respectively.

According to the boundary line responses of seed yield to the mean growing season (VE–R7), the optimum temperature for potential yield was estimated to be 26.2°C and 25.0°C for Sinpaldalkong and Daewonkong cultivars, respectively (Fig 5). The lower optimum temperature for potential seed yield in the Daewonkong large-seeded cultivar may be attributable to its lower optimum temperature for single seed weight. To evaluate the validity of these estimates, seed yields for the same experimental sets in this study were simulated using the CROPGRO-Soybean [39, 51] model; the simulated seed yields were subjected to boundary line analyses in response to growing season (VE–R7) temperatures (Fig 7). The optimum temperature ranges during the VE–R7 period for seed yield were estimated to be 26.3°C and 25.8°C for the Sinpaldalkong and Daewonkong cultivars, respectively. The simulated yield response to temperature coincided very closely with the experimental results for the Sinpaldalkong cultivar (Figs 5C and 6), whereas the simulated yield responses to temperature differed slightly from the experimentally estimated responses, where an optimum temperature of 0.8°C higher was reported for the simulated Daewonkong cultivar response. This difference may be a result of employing the same seed growth rate response to the growth temperature during the grain filling period set in the CROPGRO-Soybean model, regardless of genotype. Further studies should be conducted to explain this discrepancy.

Considering previous studies and the boundary line analysis of our four-year experiment under elevated air temperatures, it is concluded that the current air temperatures in South Korea remain below the optimum level for potential soybean yields. As the current mean air temperatures during the soybean growing season (VE–R7) ranges from 21.7 (in the north) to 24.6°C (in the south) in South Korea, further warming of only 1°C may have adverse effects on the potential yield of Daewonkong cultivar in the southern regions. However, on the whole, temperature rise of less than approximately 1°C would not become a critical limiting factor for soybean production in South Korea.

## Supporting Information

S1 Dataset Daily mean air temperatures in the experimental greenhouses that were controlled to target temperatures of AT, AT+1.5°C, AT+3.0°C, and AT+5.0°C during the growing seasons in 2009, 2013, 2014, and 2015. (XLSX)

S2 Dataset Phenology responses to temperature treatments of the Sinpaldalkong and Daewonkong cultivars planted during the typical sowing season (mid-June). (XLSX)

S3 Dataset Responses of seed yield and yield components to temperature treatments that were controlled to target temperatures of AT, AT+1.5°C, AT+3.0°C, and AT+5.0°C in two determinate soybean cultivars with different maturity groups (MGs) planted during the typical sowing season (mid-June). (XLSX)

S4 Dataset Relations between seed yield and yield components of the Sinpaldalkong and Daewonkong cultivars grown under elevated temperature conditions with different sowing dates over a four-year period. (XLSX)

S5 Dataset Boundary line analysis of seed number, single seed weight, and seed yield responses to mean air temperatures during specific growth periods for two determinate soybean cultivars, Sinpaldalkong and Daewonkong. The potential yields were calculated by multiplying the boundary points of the seed number by the single seed weight for the Sinpaldalkong and Daewonkong cultivars, respectively.
(XLSX)

S6 Dataset The numbers of flowers, pods, and the pod set ratio presented as relative values to the AT for the Sinpaldalkong and Daewonkong cultivars, grown under four different temperature conditions in 2015. (XLSX)

S7 Dataset Simulated soybean yields and yield components using the CROPGRO-Soybean model for Sinpaldalkong and Daewonkong cultivars, grown under elevated temperatures with different sowing dates over a four- year period. (XLSX)

S1 Appendix Regression analyses for the development rate during the VE–R1, R1–R5, and R5–R7 periods of the Sinpaldalkong and Daewonkong cultivars planted during the typical sowing season (mid-June). (DOCX)

S2 Appendix ANOVA analysis for the temperature treatment and cultivar and Duncan's multiple range tests for the temperature treatment on phenology of the Sinpaldalkong and Daewonkong cultivars planted during the typical sowing season (mid-June). (DOCX)

S3 Appendix ANOVA analysis for the temperature treatment and cultivar and Duncan's multiple range tests for the temperature treatment on yield and its components of the Sinpaldalkong and Daewonkong cultivars planted during the typical sowing season (mid-June). (DOCX)

S4 Appendix Correlation and path analyses for yield and yield components in the Sinpaldalkong and Daewonkong cultivars grown under elevated temperature conditions with different sowing dates over a four-year period. (DOCX)

S5 Appendix Quantile regression analysis of seed number, single seed weight, and seed yield responses to mean air temperatures during specific growth periods for two determinate soybean cultivars, Sinpaldalkong and Daewonkong. (DOCX)

S6 Appendix ANOVA analysis for the temperature treatment and cultivar and Duncan's multiple range tests for the temperature treatment on the numbers of flowers, pods, and the pod set ratio of the Sinpaldalkong and Daewonkong cultivars, grown under four different temperature conditions in 2015. (DOCX)

S7 Appendix Quantile regression analysis of simulated yield using the CROPGRO-Soybean model to mean air temperatures during the VE-R7 periods for Sinpaldalkong and Daewonkong cultivars, grown under elevated temperatures with different sowing dates over a four- year period. (DOCX)
