# Supplementary material for: Phenology and Seed Yield Performance of Determinate Soybean Cultivars Grown at Elevated Temperatures in a Temperate Region
Source: PLoS One. 2016 Nov 3;11(11):e0165977. doi: 10.1371/journal.pone.0165977 (PMC5094742; doi:10.1371/journal.pone.0165977)

1. Quadratic regression analysis for the development rate during VE-R1 in the Sinpaldalkong


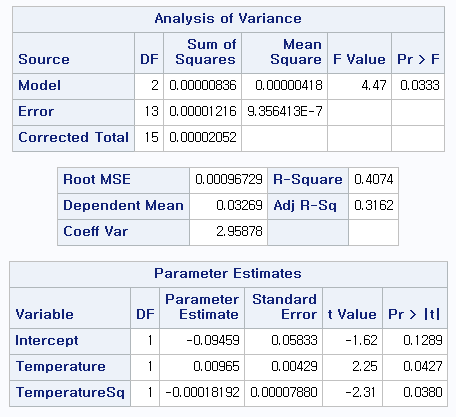


2. Quadratic regression analysis for the development rate during VE-R1 in the Daewonkong


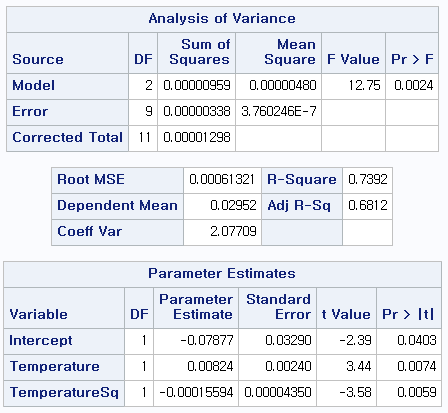


3. Linear regression analysis for the development rate during R1-R5 in the Sinpaldalkong


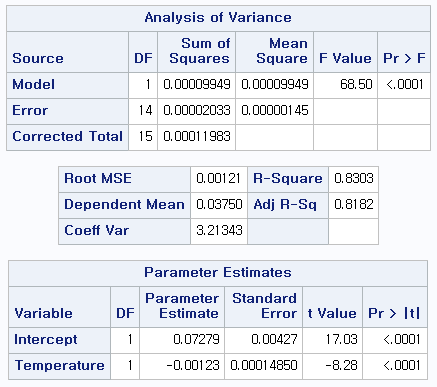


4. Linear regression analysis for the development rate during R1-R5 in the Daewonkong


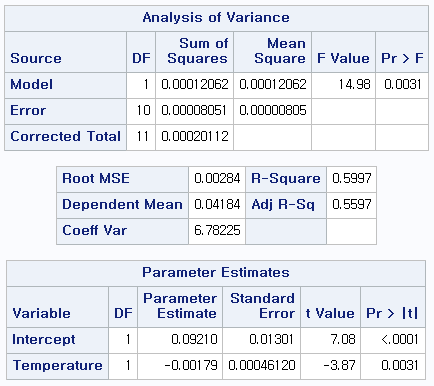


5. Linear regression analysis for the development rate during R5-R7 in the Sinpaldalkong


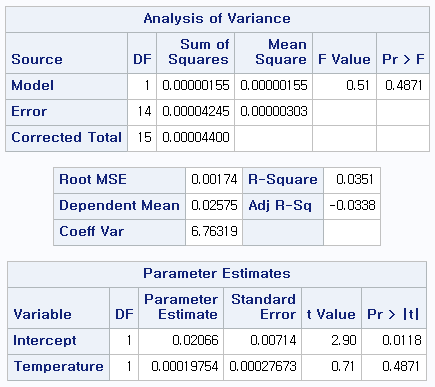


6. Linear regression analysis for the development rate during R5-R7 in the Daewonkong


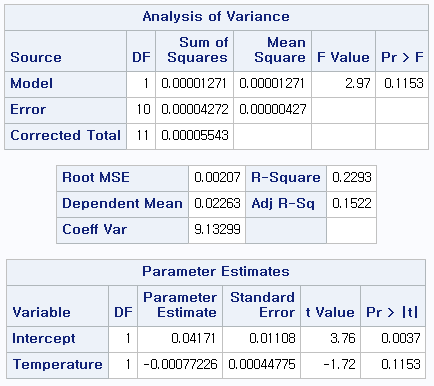

Supplement: S1 Appendix — (DOCX) [file pone.0165977.s008.docx]
