# Supplementary material for: Phenology and Seed Yield Performance of Determinate Soybean Cultivars Grown at Elevated Temperatures in a Temperate Region
Source: PLoS One. 2016 Nov 3;11(11):e0165977. doi: 10.1371/journal.pone.0165977 (PMC5094742; doi:10.1371/journal.pone.0165977)

1. ANOVA analysis for the temperature treatment and cultivar on phenology of both the Sinpaldalkong and Daewonkong


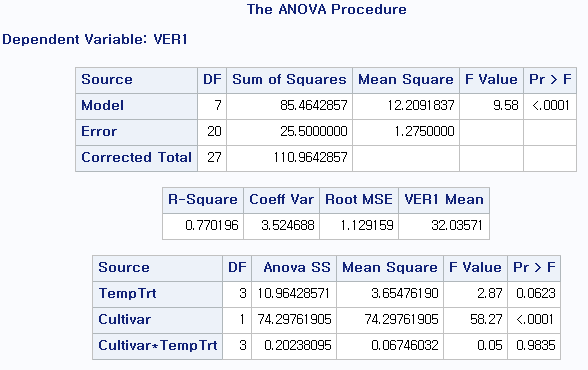

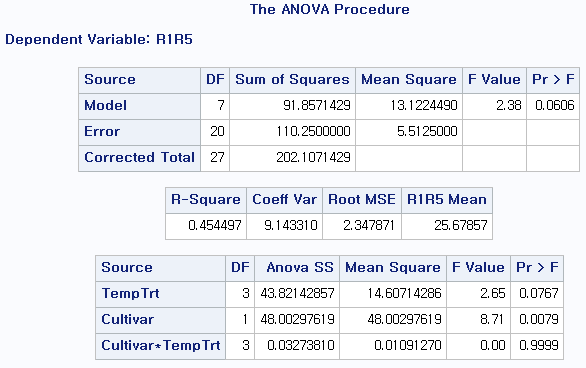

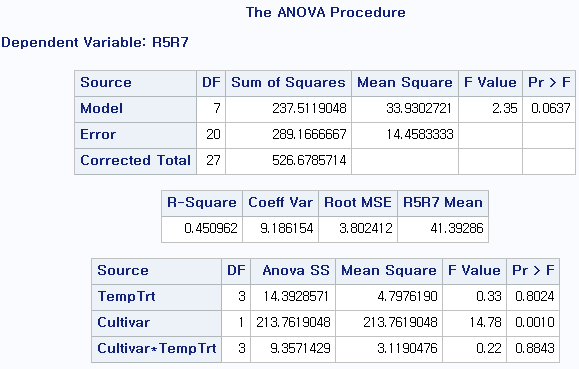

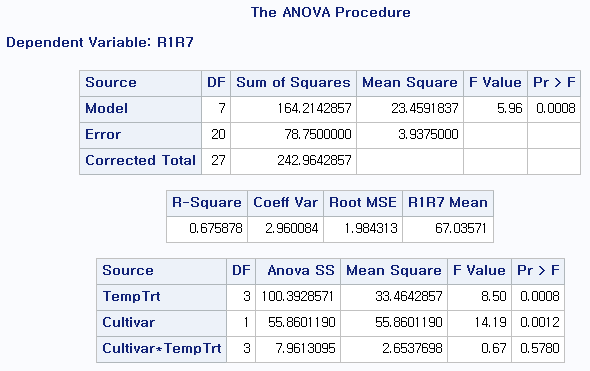

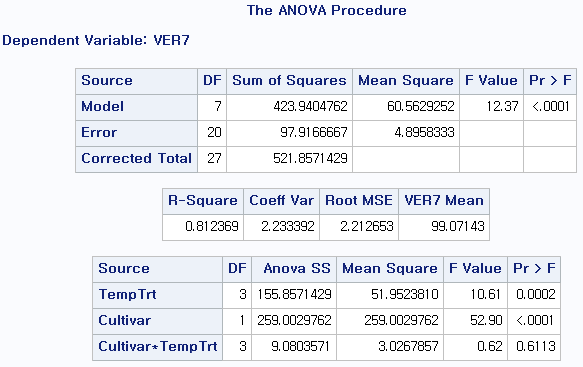


2. Duncan’s multiple range test for the temperature treatment on phenology of both the Sinpaldalkong and Daewonkong


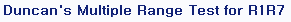


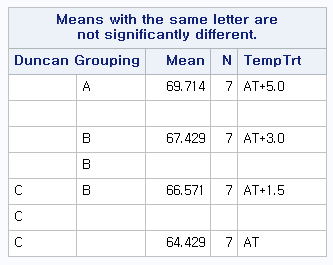


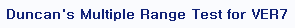


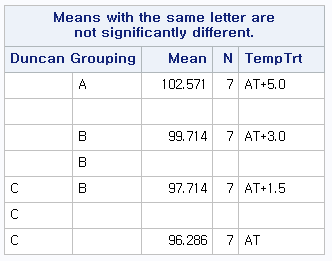


3. Duncan’s multiple range test for the temperature treatment on phenology of the Sinpaldalkong


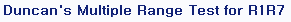


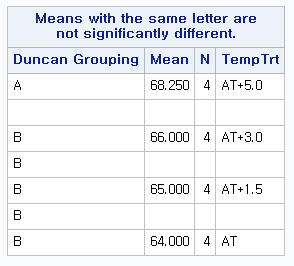


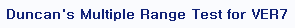


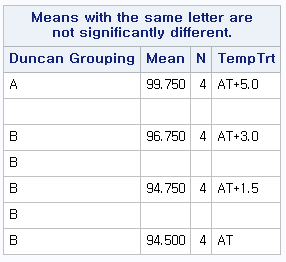


4. Duncan’s multiple range test for the temperature treatment on phenology of the Daewonkong


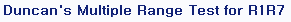


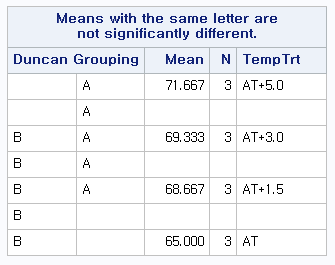


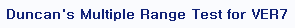


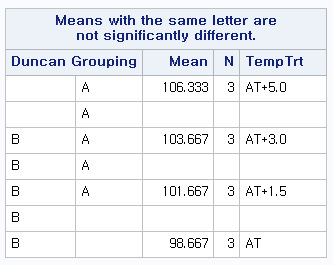

Supplement: S2 Appendix — (DOCX) [file pone.0165977.s009.docx]
