# Supplementary material for: Phenology and Seed Yield Performance of Determinate Soybean Cultivars Grown at Elevated Temperatures in a Temperate Region
Source: PLoS One. 2016 Nov 3;11(11):e0165977. doi: 10.1371/journal.pone.0165977 (PMC5094742; doi:10.1371/journal.pone.0165977)

1. Correlation analysis for yield and yield components of the Sinpaldalkong


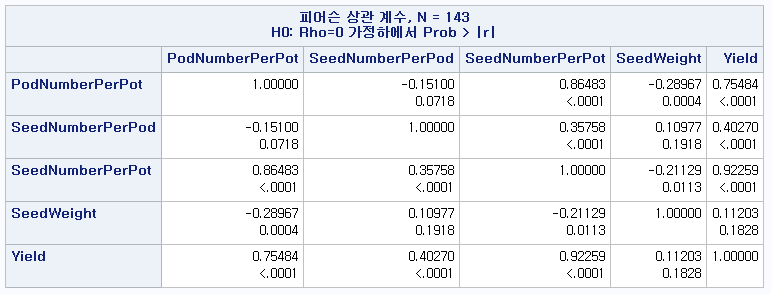


2. Correlation analysis results yield and yield components of the Daewonkong


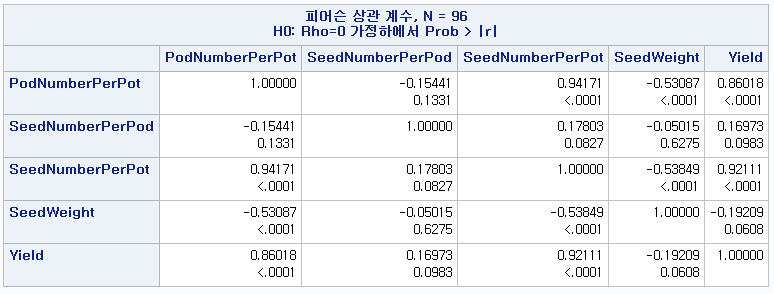


3. Path analysis for yield and yield components of the Sinpaldalkong


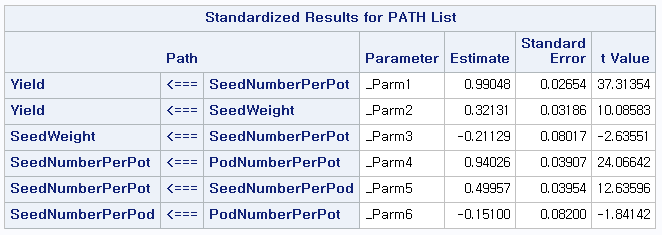

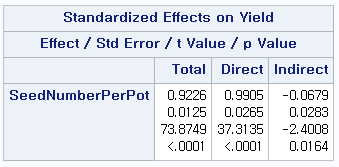

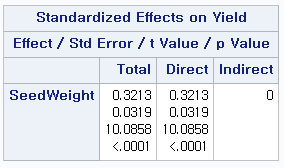

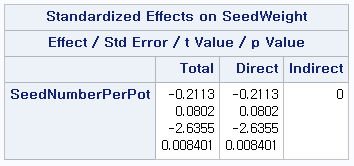

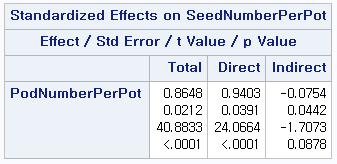

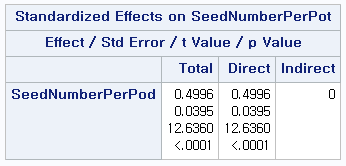

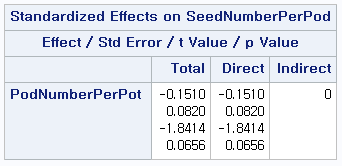


4. Path analysis for yield and yield components of the Daewonkong


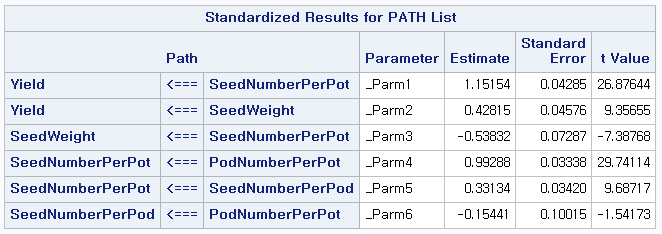

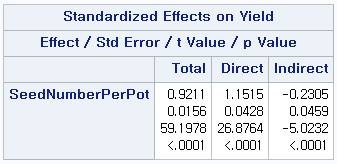

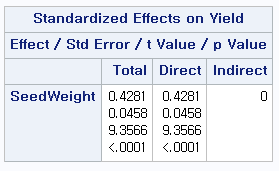

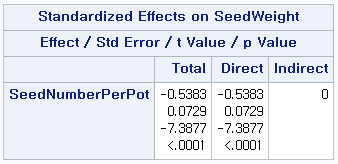

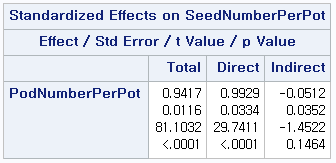

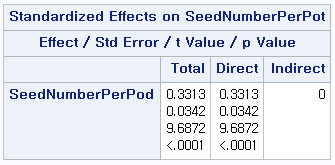

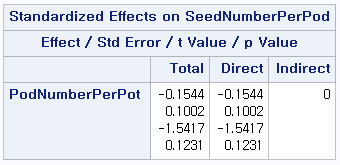

Supplement: S4 Appendix — (DOCX) [file pone.0165977.s011.docx]
