# Supplementary material for: Phenology and Seed Yield Performance of Determinate Soybean Cultivars Grown at Elevated Temperatures in a Temperate Region
Source: PLoS One. 2016 Nov 3;11(11):e0165977. doi: 10.1371/journal.pone.0165977 (PMC5094742; doi:10.1371/journal.pone.0165977)

1. 95% Quantile regression analysis for seed number in the Sinpaldalkong


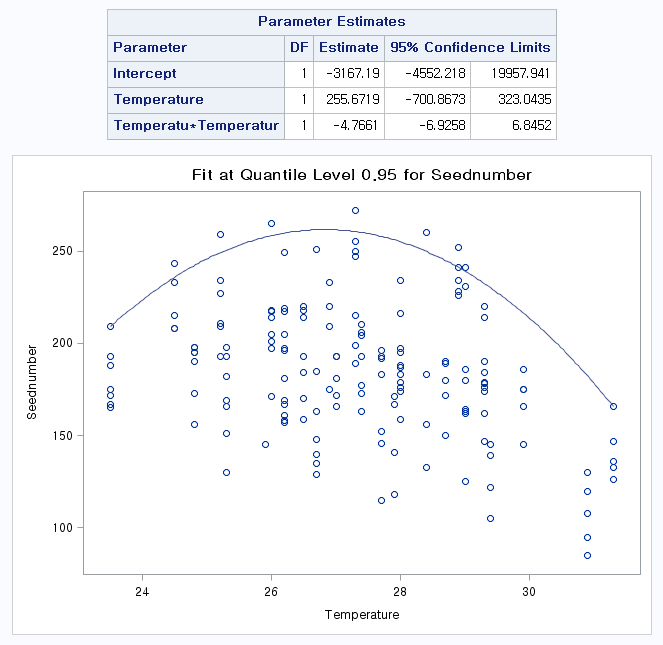


2. 95% Quantile regression analysis for seed number in the Daewonkong


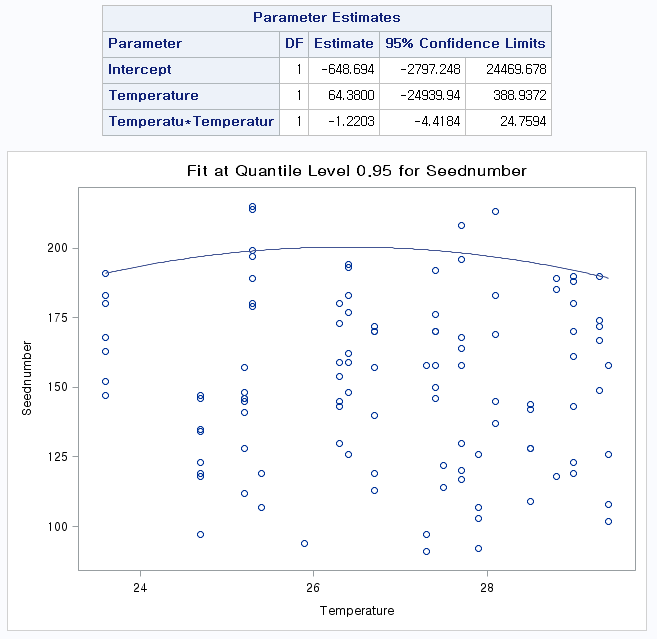


3. 95% Quantile regression analysis for single seed weight in the Sinpaldalkong


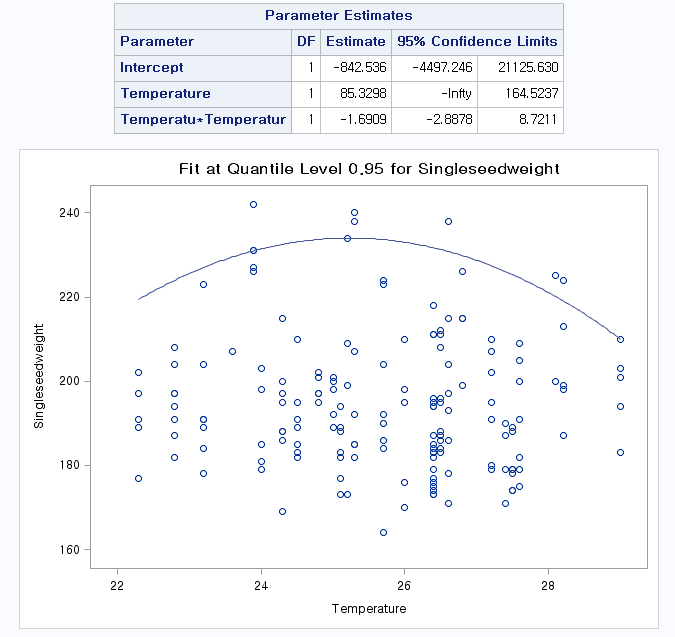


4. 95% Quantile regression analysis for single seed weight in the Daewonkong


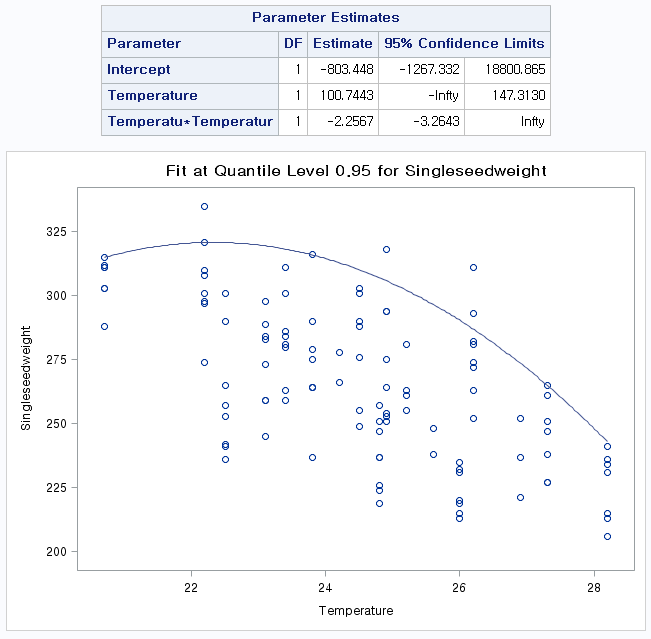

Supplement: S5 Appendix — (DOCX) [file pone.0165977.s012.docx]
