# Supplementary material for: Phenology and Seed Yield Performance of Determinate Soybean Cultivars Grown at Elevated Temperatures in a Temperate Region
Source: PLoS One. 2016 Nov 3;11(11):e0165977. doi: 10.1371/journal.pone.0165977 (PMC5094742; doi:10.1371/journal.pone.0165977)

1. 95% Quantile regression analysis for simulated yield from CROPGRO-Soybean in the Sinpaldalkong


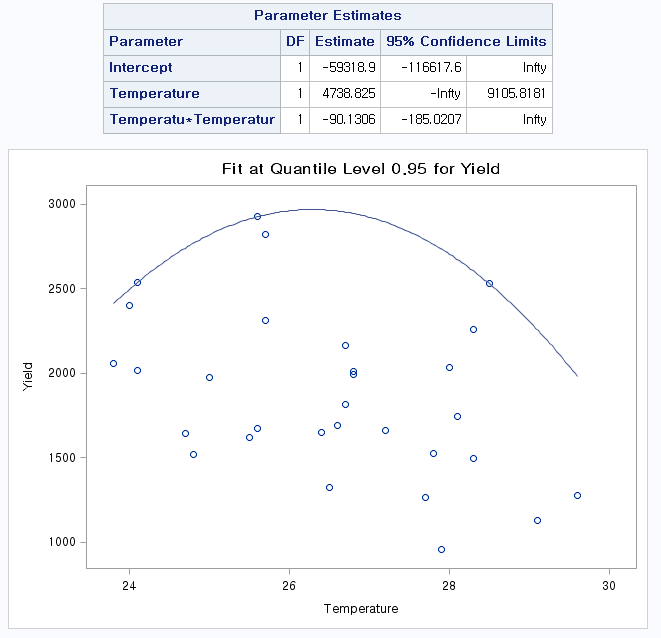


2. 95% Quantile regression analysis for simulated yield from CROPGRO-Soybean in the Daewonkong


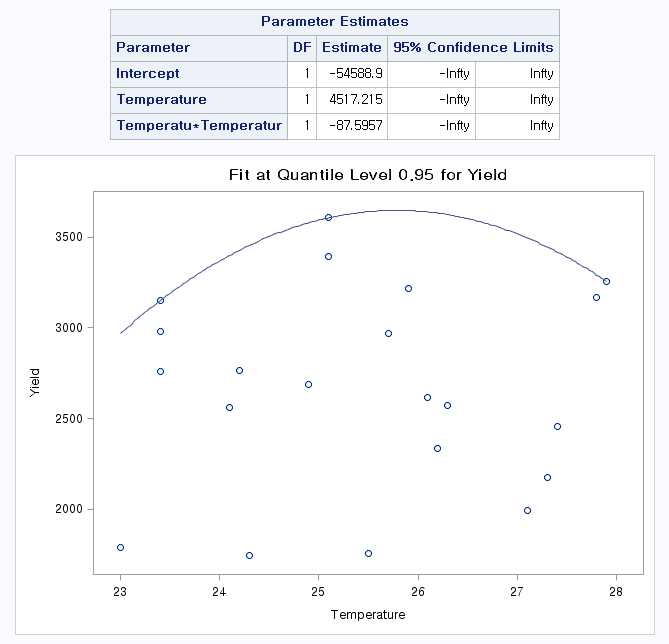

Supplement: S7 Appendix — (DOCX) [file pone.0165977.s014.docx]
